# Toward the application of metallothionein inducers as a method of prevention of air pollutant-dependent lung injury

**DOI:** 10.3389/fpubh.2025.1652263

**Published:** 2025-09-03

**Authors:** Ken-ichiro Tanaka

**Affiliations:** Laboratory of Bio-Analytical Chemistry, Faculty of Pharmacy, Research Institute of Pharmaceutical Sciences, Musashino University, Nishitokyo, Tokyo, Japan

**Keywords:** metallothionein, antioxidant, endogenous protein, oxidative stress, PM_2.5_, lung injury

## Abstract

Although Sustainable Development Goal 3.9 states the global goal of reducing air pollution-related death and disease, innovative ways to reduce air pollution-related deaths and diseases have not yet been established. To address this issue, we have established animal models using urban air dust containing the major air pollutants, suspended particulate matter (PM_10_) and fine particulate matter (PM_2.5_), and are promoting research to discover protective factors in the respiratory tract where air pollutants first reach the human body. Therefore, in this Perspective manuscript, we provide an overview of our research on the preventive effects of the endogenous protein metallothionein on air pollutant-dependent lung injury and prospects for the clinical application of metallothionein inducers. We hope that this manuscript will help to solve health hazards caused by air pollution.

## 1 Health hazards caused by air pollution

The increase in the number of deaths and diseases associated with air pollution worldwide is a major problem. Alarmingly, the annual global death toll from air pollution has risen to more than 8.8 million, exceeding the annual global death toll from smoking (1). The global goal of reducing air pollution-related deaths and diseases is clearly stated in Sustainable Development Goals 3.9 (2). Unfortunately, innovative ways to reduce deaths and diseases associated with air pollution have not yet been established. Suspended particulate matter (PM_10_) and fine particulate matter (PM_2.5_) are the main health hazards caused by air pollution. Because these particles are very small (less than 10 micrometers in diameter), they remain in the air for long periods of time, easily pass through the human airways, and reach the alveoli at the periphery of the lungs, causing various respiratory and cardiovascular diseases (1, 3). Previous studies have suggested that the induction of oxidative stress, such as the production of reactive oxygen species (ROS), is associated with exposure to these air pollutants (e.g., PM_2.5_ and PM_10_) and is an important contributing factor in respiratory diseases such as chronic obstructive pulmonary disease (COPD), asthma, pulmonary fibrosis, and lung cancer, as well as cardiovascular diseases such as heart failure, myocardial infarction, and stroke (4, 5). Therefore, we are conducting research to analyze the molecular mechanism of injury in bronchioles and alveoli, the first sites in the human body where air pollutants reach, and to discover substances that can prevent such injury. We believe that this will lead to the establishment of preventive methods against the health hazards caused by air pollution.

## 2 Overview of our previous studies

In our research to date, we have created a mouse model of acute lung injury and a cell experiment system using urban aerosol particulate matter, 99% of which is composed of particles with a diameter of 10 μm or less [purchased from the National Institute for Environmental Studies (Tsukuba, Japan)] (6), and are conducting research using these models. We also used these models to analyze the function of protective factors that may prevent respiratory diseases caused by air pollutants. In our first study on air pollution, we analyzed the effectiveness of human serum albumin-fused thioredoxin (HSA-Trx), a drug delivery system (DDS) formulation that improves the *in vivo* stability of the endogenous antioxidant protein, thioredoxin (7). Using an *in vivo* imaging system, we found that HSA-Trx significantly suppressed neutrophil-associated inflammatory responses, such as neutrophil extracellular traps and acute lung injury, by suppressing ROS production in mouse lungs caused by urban aerosols. This first study showed that suppressing oxidative stress such as ROS production caused by air pollutants is important for preventing respiratory diseases caused by air pollutants (8). In fact, other clinical studies and analyses using animal and cell models have also pointed out that oxidative stress, including ROS production, may be the main cause of health hazards caused by air pollutants (9–12).

In contrast, we are conducting research to analyze the protective function of metallothionein, an endogenous metal-binding protein, against various respiratory diseases and to elucidate its molecular mechanisms. Metallothionein is present in all animal cells and can bind up to 7–12 metal ions; thus, it plays a role in maintaining the homeostasis of essential trace elements and detoxifying heavy metal elements such as cadmium (13). In addition, metallothionein has approximately 20 thiol groups per molecule, which not only bind to excess heavy metals for detoxification but also react with ROS to exert an antioxidant effect (14–16). Four isoforms of metallothionein have been identified, of which metallothionein-1 and -2 are expressed in most tissues and play a role in heavy metal detoxification and antioxidant functions (13). Because of these physiological activities of metallothionein, there have been many efficacy analyses of metallothionein for liver and kidney injuries (17–19), but there have not been many analyses of metallothionein for respiratory diseases such as COPD and pulmonary fibrosis. Based on this background, we have conducted functional analysis of metallothionein in various respiratory diseases, and have found that porcine pancreatic elastase-induced lung injury and cigarette smoke-induced lung injury, which are animal models of COPD, are more pronounced in metallothionein-knockout (MT-KO) mice (129/Sv-MT1MT2*^tm1Bri^*) than in wild-type mice (129/SvCPJ) (20, 21). In another study, we found that treatment of alveolar epithelial cells with polaprezinc or zinc acetate induced metallothioneins in the cells, and that this induction prevented cell injury in the alveolar epithelium caused by cadmium, a heavy metal found in tobacco smoke (22). These results indicate a new function of metallothionein that has a preventive effect against the onset and exacerbation of COPD and suggest that compounds such as zinc preparations that have the ability to induce metallothionein may be a preventive method for COPD and other respiratory diseases.

**Figure 1:**
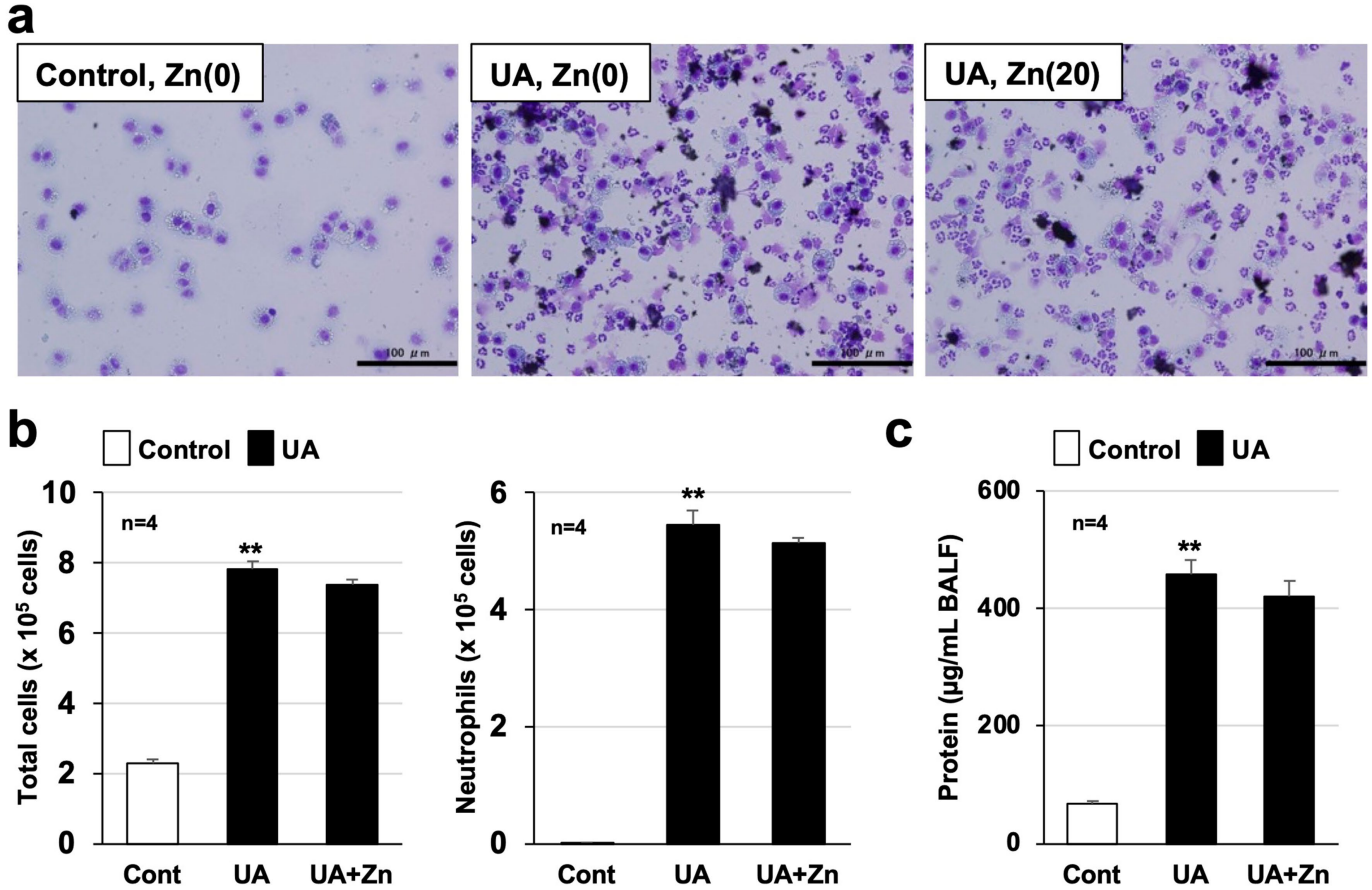
Zinc acetate had little effect on metallothionein-knockout (MT-KO) mice. Metallothionein-knockout (MT-KO) mice received a single intraperitoneal dose of sterile saline (control or urban aerosols alone group) or zinc acetate (20 mg/kg) at 0 h. Twenty-four hours after intraperitoneal administration, MT-KO mice received a single intratracheal dose of sterile saline (Cont) or an urban aerosol particle suspension (UA or UA + Zn, 1.0 mg/60 μL sterile saline per mouse). Twenty-four hours after intratracheal administration, bronchoalveolar lavage fluid (BALF) was collected from mice; cells present in the BALF were applied to slides using a Cytospin 4 cell centrifuge and stained with Diff-Quik reagent, and visualized under a light microscope (scale bar, 100 μm) **(a)**. Total cell counts were determined using a hemocytometer and neutrophil counts were calculated based on Diff-Quik staining (*n* = 4) **(b)**. Protein levels in BALF were measured using Bradford reagent (*n* = 4) **(c)**. Values are the mean ± SEM; **p < 0.01. (**, vs. Cont, one-way analysis of variance followed by Dunnett’s test). The results showing that pre-administration of zinc acetate suppresses air pollutant-dependent acute lung injury in wild-type mice have already been published in *Biomedicine and Pharmacotherapy* (23). In contrast, pre-administration of zinc acetate showed little efficacy in MT-KO mice (in this figure). These results strongly support the notion that metallothionein inducers prevent air pollutant-dependent acute lung injury.

**SCHEME 1:**
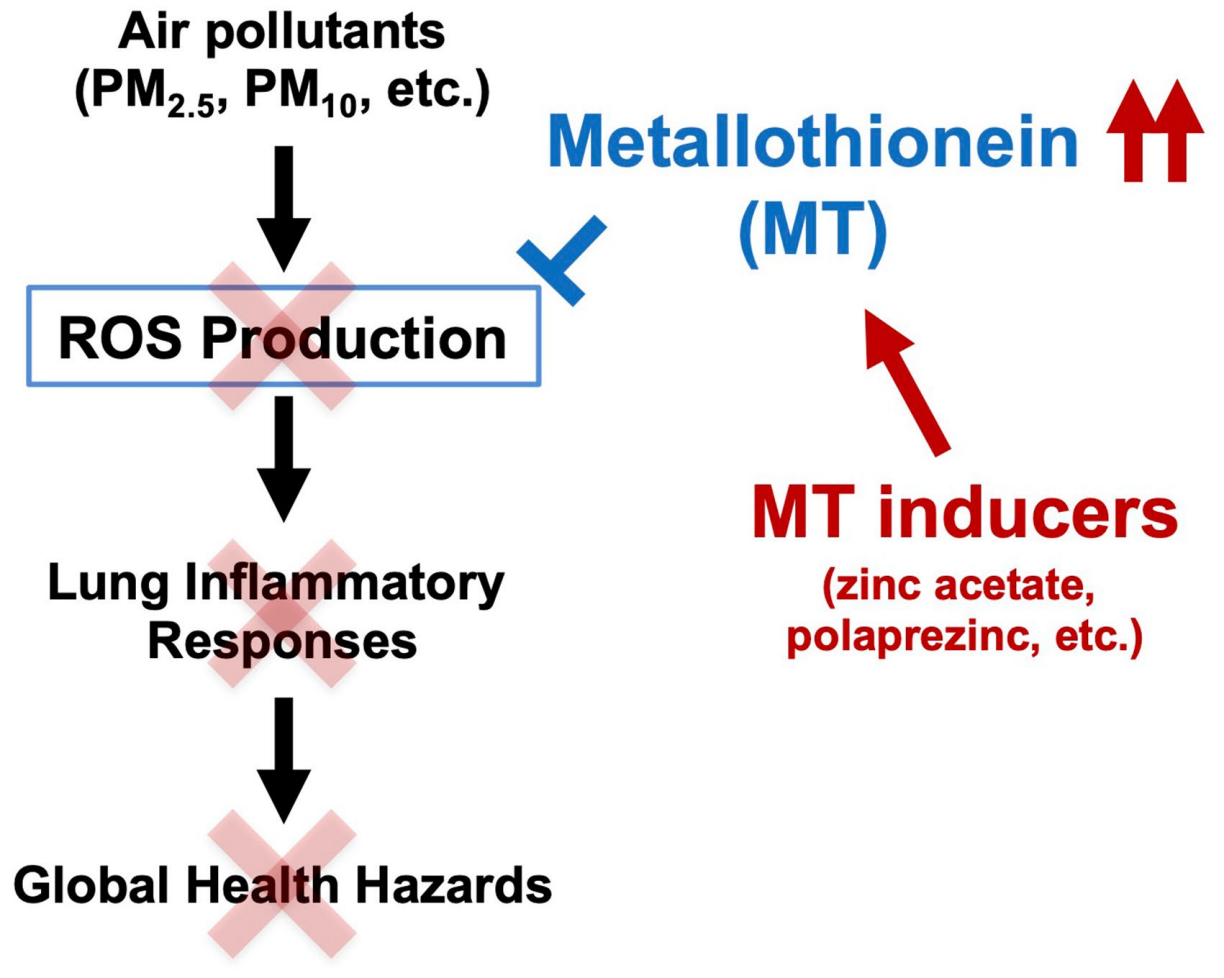
Mechanisms of acute lung injury induced by air pollutants and preventive effects of metallothionein. Metallothionein inducers, such as zinc acetate or polaprezinc, exert their antioxidant effects via induction of metallothionein in the mouse lung, ultimately preventing acute lung injury caused by air pollutants.

Because air pollutants such as PM_2.5_ contain metal elements such as aluminum, iron, magnesium, and titanium (6), we thought it was important to clarify the relationship between metallothionein, a metal-binding protein, and lung injury caused by air pollutants. Thus, we recently analyzed the efficacy of metallothionein against air pollutant-dependent acute lung injury in MT-KO mice to identify novel functions of metallothionein (23). As described below, in part in the results published in *Biomedicine and Pharmacotherapy*, we found that acute lung injury caused by urban aerosols was significantly exacerbated in MT-KO mice compared to that in wild-type mice due to enhanced lung ROS production. ROS are known to activate NF-kB, a transcription factor that promotes inflammatory responses induced by various stimuli (24). Thus, we speculate that excessive ROS production in MT-KO mice may have promoted the NF-kB-mediated inflammatory responses in the lungs. Furthermore, we found that the administration of zinc acetate (20 mg/kg) to wild-type mice markedly enhanced metallothionein induction (*Mt1* and *Mt2* mRNA) in the lungs, and that this induction significantly suppressed acute lung injury caused by urban aerosols. In contrast, as shown in Figure 1, pre-administration of zinc acetate (20 mg/kg) to MT-KO mice hardly suppressed the increase in total inflammatory cell counts, neutrophil counts, and protein levels in the bronchoalveolar lavage fluid caused by urban aerosols. These results strongly support our hypothesis in *Biomedicine and Pharmacotherapy* that the preventive effect of zinc acetate on air pollutant-dependent acute lung injury is mediated by metallothionein induction. In other words, we suggest that compounds with metallothionein-inducing properties, such as zinc acetate, are promising candidates for the prevention of air pollution-induced health hazards (Scheme 1).

## 3 Future perspective of our study

To establish metallothionein inducers as a method for preventing health hazards caused by air pollution, several issues remain to be resolved, and we are currently attempting to resolve them. First, if zinc acetate is to be applied as a method of preventing health hazards caused by air pollution, it is necessary to discover the optimal method of administration. In our recent study, we conducted an efficacy analysis of intraperitoneal administration of zinc acetate (23); however, its clinical application in the intraperitoneal setting is not very realistic. The results of clinical trials investigating the pharmacokinetics of oral zinc acetate have already been reported. Following the administration of 50 mg of zinc acetate to healthy adult males, the AUC_0–24 h_ was 672.44 ± 165.38 μg•h/dL and the Cmax was 144.67 ± 31.11 μg/dL (https://pins.japic.or.jp/pdf/newPINS/00071288.pdf). Although inhaled zinc acetate has not yet been clinically applied, it is simple and can deliver zinc acetate directly to the respiratory tract damaged by air pollutants. Therefore, we believe that these administration methods are clinically feasible and that the efficacy of zinc acetate should be analyzed using these methods. In addition, we also need to clarify in pre-clinical studies the side effects that may occur with prolonged clinical use of zinc acetate in these dosing regimens. It is well known that insufficient zinc intake in humans can cause health problems, but excessive zinc intake can cause acute health problems, such as nausea, vomiting, loss of appetite, abdominal cramps, diarrhea, and headaches (25). Moreover, chronic zinc intake is associated with adverse effects on urinary physiology (26). We believe that the effectiveness of oral or inhalation administration of zinc acetate in the treatment of air pollutant-dependent lung injury, while also conducting safety studies on zinc acetate, is likely to lead to practical applications. In addition, as previous studies have only examined zinc acetate administration before treatment, it is important to analyze the efficacy of zinc acetate administration after exposure to urban aerosols in future studies.

Furthermore, our model of air pollutant-dependent acute lung injury has limitations. In our previous studies, acute lung injury was assessed 24–48 h after a single intratracheal administration of urban aerosols (8, 23, 27). People living in areas with severe air pollution are repeatedly exposed to air pollutants over long periods, which can lead to devastating health consequences. In particular, long-term exposure to air pollutants has been reported to induce respiratory symptoms similar to the clinical manifestations of COPD, pneumonia, and asthma (28–32). Our model of acute lung injury due to a single exposure to air pollutants is very useful for people who travel to areas with severe air pollution in the short term. However, to solve global health problems, it is important to establish animal and cellular models for long-term repeated exposure to air pollutants and to analyze the efficacy of metallothionein inducers, such as zinc acetate, based on indicators such as the degree of inflammatory response, tissue damage, and respiratory dysfunction. We are convinced that such efforts will lead to a solution for global health problems.

## 4 Potential clinical application of MT inducers other than zinc acetate

It has been reported that various metals other than zinc, such as cadmium and iron, induce metallothionein through pathways mediated by metal regulatory transcription factor 1 (MTF-1) and metal responsive element (MRE). In addition, it has been reported that ROS, glucocorticoids, and various cytokines induce metallothionein through antioxidant responsive element (ARE), glucocorticoid responsive element (GRE), and activator protein 1 (AP-1) sites (14–16). Our research group is focusing on selenium as a metal other than zinc. Selenium is an essential trace element present in the active centers of antioxidant enzymes, such as glutathione peroxidase (GPX) and thioredoxin reductase (TrxR) (33, 34). Selenium has been reported to alleviate cadmium-induced cerebellar injury by activating metal responses, including metallothionein induction via MTF-1, in both *in vivo* and *in vitro* models (35). For these reasons, we speculate that selenium, like zinc, may be effective in preventing air pollutant-dependent lung injury via metallothionein induction, and we look forward to further research developments. We would argue that establishing metallothionein inducers, such as zinc and selenium, as a method of preventing health problems caused by air pollution would be one of the major contributions to public health and would also help reduce healthcare costs in an aging society.

## 5 Limitations of our previous study

The animal model used in previous studies was a model of acute lung injury caused by a single dose of urban aerosol. In future studies, it is extremely important to establish a chronic lung injury model resulting from multiple doses of urban aerosols and analyze the preventive effects of metallothionein.

## Data Availability

The original contributions presented in the study are included in the article/supplementary material, further inquiries can be directed to the corresponding author.
